# Preparation of 6-Amino-N-hydroxyhexanamide-Modified Porous Chelating Resin for Adsorption of Heavy Metal Ions

**DOI:** 10.3390/polym16141966

**Published:** 2024-07-09

**Authors:** Shaomin Liu, Zihan Wang, Mingyi He, Jinglin Zhu

**Affiliations:** 1 School of Earth and Environment, Anhui University of Science and Technology, Huainan 232001, China; zihanwang1999@163.com (Z.W.); 18116779416@163.com (M.H.); jlzhu@aust.edu.cn (J.Z.); 2 State Key Laboratory of Mining Response and Disaster Prevention and Control in Deep Coal Mines, Anhui University of Science and Technology, Huainan 232001, China

**Keywords:** heavy metal ions, adsorption, hydroxamic acid, chelating resin, modification

## Abstract

The pollution of water bodies by heavy metal ions has recently become a global concern. In this experiment, a novel chelating resin, D851-6-AHHA, was synthesized by grafting 6-amino-N-hydroxyhexanamide (6-AHHA) onto the (-CH_2_N-(CH_2_COOH)_2_) group of the D851 resin, which contained a hydroxamic acid group, amide group, and some carboxyl groups. This resin was developed for the purpose of removing heavy metal ions, such as Cr(III) and Pb(II), from water. The findings from static adsorption experiments demonstrated the remarkable adsorption effectiveness of D851-6-AHHA resin towards Cr(III) and Pb(II). Specifically, the maximum adsorption capacities for Cr(III) and Pb(II) were determined to be 91.50 mg/g and 611.92 mg/g, respectively. Furthermore, the adsorption kinetics of heavy metal ions by D851-6-AHHA resin followed the quasi-second-order kinetic model, while the adsorption isotherms followed the Langmuir model. These findings suggest that the adsorption process was characterized by monolayer chemisorption. The adsorption mechanism of D851-6-AHHA resin was comprehensively investigated through SEM, XRD, FT-IR, and XPS analyses, revealing a high efficiency of D851-6-AHHA resin in adsorbing Cr(III) and Pb(II). Specifically, the (-C(=O)NHOH) group exhibited a notable affinity for Cr(III) and Pb(II), forming stable multi-elemental ring structures with them. Additionally, dynamic adsorption experiments conducted using fixed-bed setups further validated the effectiveness of D851-6-AHHA resin in removing heavy metal ions from aqueous solutions. In conclusion, the experimental findings underscored the efficacy of D851-6-AHHA resin as a highly efficient adsorbent for remediating water bodies contaminated by heavy metal ions.

## 1. Introduction

Environmental pollution has become a pervasive global concern due to its far-reaching harmful effects on ecosystems, and heavy metals pose a significant threat to aquatic ecosystems due to their limited degradability, high toxicity, and easy accumulation [1,2]. Heavy metals are gradually enriched along the ecological food chain, posing a risk of severe health complications even at exceptionally low concentrations. These potential afflictions include cancer, cumulative poisoning, cerebral impairment, and neurological disorders [3,4,5]. For example, Cd (II) has the potential to trigger renal dysfunction, kidney lesions, and even the destruction of erythrocytes, consequently exerting adverse effects on physiological systems [6,7,8]. Therefore, researchers must seek more strategies to mitigate the condition of heavy metal ion pollution. Currently, a variety of technologies have emerged in the field of heavy metal ion removal from wastewater, encompassing methodologies such as chemical precipitation, ion exchange, membrane separation, and bioremediation [9,10,11]. Among these methodologies, adsorption is widely recognized as one of the most efficient and economical techniques for the extraction of heavy metal ions due to its simplicity, relatively modest cost, and practicality [12,13,14]. Consequently, a variety of adsorbents, including activated carbon, starch, and calcium carbonate, have been employed to extract heavy metal ions from water bodies [12]. However, the adsorption efficiency of many adsorbents is not ideal. Taking starch as an example, it not only exhibits a low adsorption capacity, but also has challenges in recycling and reuse, making it susceptible to secondary pollution. Hence, there remains significant potential for the development of adsorbents with a high adsorption capacity, facile separation, and efficient regeneration in the field of heavy metal wastewater treatment [15,16].

Due to the presence of diverse coordination groups on their surfaces, chelating resins exhibit a notable adsorption capacity for specific heavy metal ions. [17,18]. These groups can effectively establish multi-element rings with heavy metal ions, helping to enhance adsorption capabilities. Therefore, the modification of chelating resin through grafting functional groups can enhance its adsorption capacity [19].

The isohydroxamic acid moiety (-C(=O)NHOH) is generally considered a derivative of carboxylic acids. The nitrogen (N) and oxygen (O) atoms within this moiety possess adjacent lone electron pairs, which endows the isohydroxamic acid moiety with a potent chelating capability for heavy metal ions [20]. Through chelation, the isohydroxamic acid moiety forms a five-membered ring structure with heavy metal ions, and their binding of heavy metal ions is about seven orders of magnitude higher than that of carboxylic acids [20,21]. Moreover, previous studies have also proved that the (-C(=O)NHOH) group has an excellent adsorption capacity. Duan et al. synthesized a novel poly (2-acrylamide-glutaric hydroxamic acid) resin incorporating the (-C(=O)NHOH) group, and the maximum adsorption capacities of the new resin for Cu^2+^ and Ni^2+^ were 436.08 and 195.05 mg/g, respectively [18]. Cao et al. synthesized a poly (6-acryloylamino caproxamic acid) resin through the reaction of polyacrylic acid resin with 6-amino caproxamic acid, and the adsorption capacities of the resin for the La(III), Ce(III), and Y(III) ions were 1.030, 0.962, and 1.450 mmol/g, respectively [22]. The macroporous styrenic chelating resin (D851) represents a mesoporous material with iminodiacetic acid groups (-CH_2_N-(CH_2_COOH)_2_), while the iminodiacetic acid group contains a large number of -COOH groups. Therefore, D851 resin is an ideal matrix for preparing new adsorbents [23,24]. In this study, a novel resin (D851-6-AHHA) was synthesized by grafting 6-amino-N-hydroxyhexanamide onto the (-CH_2_N-(CH_2_COOH)_2_) group of D851 resin. The synthesized D851-6-AHHA resin contains a large number of (-C(=O)NHOH) groups and partially unreacted (-CH_2_N-(CH_2_COOH)_2_) groups on its surface. These functional groups enable the new resin to efficiently remove various heavy metal ions. Subsequently, Cr(III) and Pb(II) were selected as adsorption objects, and the interaction between the D851-6-AHHA resin and these ions was systematically investigated. The impacts of pH, temperature, initial concentration, and contact time on the adsorption characteristics of the D851-6-AHHA resin were examined, and the fixed-bed adsorption experiments of Cr(III) and Pb(II) by D851-6-AHHA resin were simulated. The properties and adsorption mechanism of the D851-6-AHHA resin were analyzed using SEM-EDS, FT-IR, XRD, XPS, and BET techniques. These analyses provided a comprehensive insight into the adsorption performance of the modified resin in relation to heavy metal ions, enhancing our understanding of its adsorption capabilities.

## 2. Experiments

### 2.1. Materials and Chemicals

Macroporous styrene chelating resin (D851) was purchased from Tianjin Jinda Resin Technology Co., Ltd. (Tianjin, China). The particle size of the resin was 0.45~1.25 mm, the skeleton structure was macroporous styrene–divinylbenzene, and the functional group was iminodiacetic acid groups. Hydroxylamine hydrochloride was purchased from Shanghai Sinopharm Chemical Reagent Group. CrCl_3_, Pb(NO_3_)_2_, sodium hydroxide, hydrochloric acid, and caprolactam were purchased from Shanghai Aladdin Biochemical Technology Co., Ltd. (Shanghai, China). The above reagents were analytically pure, and the experimental water was ultrapure.

### 2.2. Preparation of 6-Amino-N-Hydroxyhexanamide (6-AHHA)

Hydroxylamine hydrochloride (13.90 g, 0.20 mol) and sodium hydroxide (16.67 g, 0.40 mol) were placed in 100 mL of ultrapure water and fully reacted for 2.0 h at 0 °C. Then, (22.63 g, 0.20 mol) caprolactam and hydroxylamine solutions were added into a three-necked flask and kept at 100 °C for 3.0 h to synthesize 6-amino-N-hydroxyhexanamide liquid (6-AHHA) [25].

### 2.3. Synthesis of D851-6-AHHA Resin

**Scheme 1:**
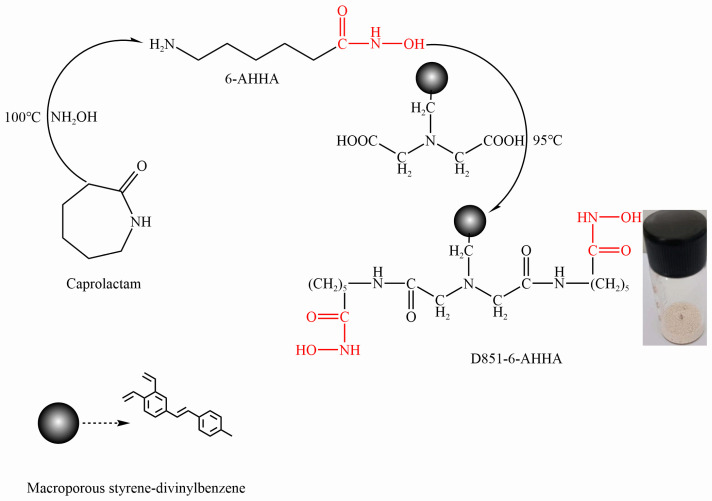
Synthesis process of D851-6-AHHA resin.

For this, 50 mL of 6-AHHA solution and 10.0 g of D851 resin were combined in a hydrothermal synthesis reactor, and the resultant mixture was maintained at 95 °C for 4.0 h. Subsequently, the mixture was cooled to room temperature, stirred, and filtered to obtain yellow D851-6-AHHA resin. The obtained resin was then dried at 45 °C for 24 h in a vacuum drying oven. The synthesis process of D851-6-AHHA resin is shown in Scheme 1.

### 2.4. Analytical Methods

The surface morphology of D851-6-AHHA resin was characterized using scanning electron microscopy (SEM, Thermo Fisher Scientific, Apreo 2C, Waltham, MA, USA). The distribution of functional groups on the adsorbent surface was evaluated using Fourier transform infrared spectroscopy (FT-IR, Thermo Scientific, Nicolet-iS10, Waltham, MA, USA). D851-6-AHHA resin before and after adsorption were analyzed by X-ray photoelectron spectroscopy (XPS) using a Thermo Fisher Scientific (Waltham, MA, USA), Escalab 250 Xi spectrometer.

### 2.5. Bath Adsorption Experiments

A specific amount of pretreated D851-6-AHHA resin was weighed in batches and placed into 250 mL conical bottles. Subsequently, 50 mL and 100 mL of Cr(III) and Pb(II) solutions with various concentrations were added to the bottles, which were placed in a thermostatic oscillator at 25 °C for 12 h. The impacts of initial solution pH, contact time, initial concentration, and temperature on D851-6-AHHA resin were examined through batch static adsorption experiments. For the regeneration of the D851-6-AHHA resin, the adsorbed resin was collected, washed with ultra-pure water to achieve neutrality, and subsequently desorbed with 0.5 mol/L HCl solution for 6.0 h. This cycle was repeated five times.

The concentration of residual heavy metal ions in the supernatant was determined by inductively coupled plasma emission spectrometry (ICP-AES, Thermo, Waltham, MA, USA). Subsequently, the equilibrium adsorption capacity and removal rate of Cr(III) and Pb(II) in the solution by the D851-6-AHHA resin were calculated using Equations (1) and (2), respectively [26].

(1) $$Q_{e}=\frac{\left(C_{0}-C_{e}\right)V}{m}$$

(2) $$R_{e}=\frac{\left(C_{0}-C_{e}\right)}{C_{0}}\times 100\%$$

where C_0_ and C_e_ represent the initial and equilibrium metal ion concentrations (mg/L), respectively, V denotes the solution volume (L), and m stands for the dry weight (g) of D851-6-AHHA resin [27].

### 2.6. Column Experiments

To further evaluate the application performance of D851-6-AHHA resin in wastewater treatment, column adsorption experiments were conducted. The concentrations of Cr(III) and Pb(II) were set to 200 mg/L and 400 mg/L, respectively. Then, 0.6 g of D851-6-AHHA resin was added to the adsorption column, and the column height was set to 2.0 cm. Simultaneously, a peristaltic pump was employed to deliver the wastewater to the adsorption column at a flow rate of 2.5 mL/min, and the effluent was collected at a certain time interval.

## 3. Results and Discussion

### 3.1. Characterization

**Figure 1:**
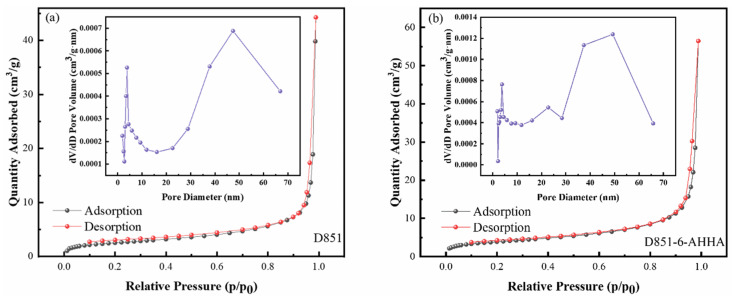
(**a**) N_2_ adsorption–desorption isotherm and pore size distribution of D851 resin, (**b**) N_2_ adsorption–desorption isotherm and pore size distribution of D851-6-AHHA resin.

The results of the BET test for the D851 and D851-6-AHHA resins are shown in Table S1 and Figure 1. The N_2_ adsorption–desorption isotherms of the two resins exhibited Type IV. Meanwhile, the H_3_ hysteresis loop emerged at a relative pressure range of 0.9~1.0. This suggested the presence of irregular mesoporous structures within these materials. The specific surface area of the D851-6-AHHA resin was measured at 14.057 m^2^∙g^−1^, which indicates a significant increase in the specific surface area over the original resin. This enhancement makes it more conducive to adsorption processes [28]. Compared to the original resin, the pore volume of the D851-6-AHHA resin has also increased from 0.068 cm^3^∙g^−1^ to 0.086 cm^3^∙g^−1^. The increase in the number of pores resulted in a decrease in the average pore size of the D851-6-AHHA resin. This change could be attributed to the modification of the functional groups, resulting in the loaded 6-AHHA occupying part of the pores of the D851 resin. Therefore, it can be deduced that the successful grafting of the 6-AHHA onto the D851 resin produced this change [29].

**Figure 2:**
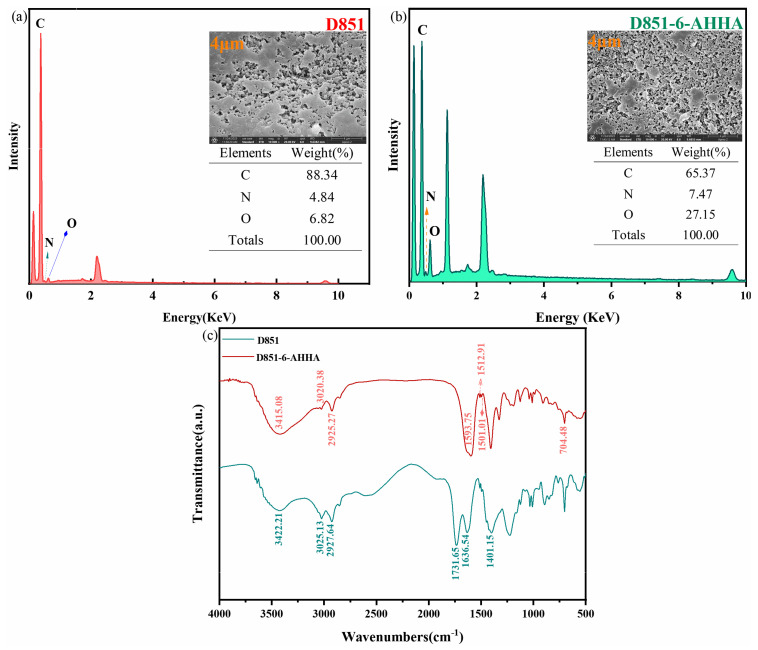
SEM-EDS images (**a**,**b**) and FT-IR spectra (**c**) of D851 and D851-6-AHHA resins.

As depicted in Figure 2a,b, the external morphology of the D851 and D851-6-AHHA resins was analyzed using SEM-EDS. It is evident that the surface of D851-6-AHHA is rougher than that of D851. Additionally, the external morphology of the D851-6-AHHA resin appears coral-like with a large number of pore holes. This characteristic facilitates the provision of ample adsorption sites for heavy metals in the solution, thereby enabling the adsorption of more heavy metal ions [5]. Compared to the EDS analysis of the D851 resin, the observed increase in the nitrogen (N) and oxygen (O) elements and the decrease in carbon (C) elements in the D851-6-AHHA resin suggest the successful grafting of 6-AHHA onto the D851 resin.

The FT-IR spectra of the D851 and D851-6-AHHA resins are illustrated in Figure 2c. For the D851 resin, the O-H stretch band of the (-CH_2_N-(CH_2_COOH)_2_) group is observed at 3422.21 cm^−1^, while the absorption peak at 1636.54 cm^−1^ corresponds to the C-N stretching vibration [30]. The sharp absorption peak at 1401.15 cm^−1^ is attributed to the vibration of CH_2_, and the peak at 1731.65 cm^−1^ is attributed to the C=O stretching band of the (-CH_2_N-(CH_2_COOH)_2_) group [31]. Additionally, the stretching vibrational peaks at 3025.13 and 2927.64 cm^−1^ represent C-H on the benzene ring. For the D851-6-AHHA resin, the O-H and N-H stretching bands of the (-CH_2_N-(CH_2_COOH)_2_) group are observed at 3415.08 and 3020.38 cm^−1^, respectively, while the C-H stretching vibrational peak is present at 2925.27 cm^−1^ [32]. The peak at 1593.75 cm^−1^ corresponds to the C=O stretching band of the (-CH_2_N-(CH_2_COOH)_2_) group [33]. Furthermore, the adsorption peaks at 1512.91 and 1501.01 cm^−1^ are attributed to the C=O and C-N stretching bands of the (-C(=O)NHOH) group, respectively, and the peak at 704.48 cm^−1^ is attributed to the (-CH_2_)_5_ vibration [34]. Therefore, the results of the FT-IR spectral analysis indicated the successful grafting of 6-AHHA onto the D851 resin.

### 3.2. Bath Adsorption Experiments

#### 3.2.1. Effect of pH

**Figure 3:**
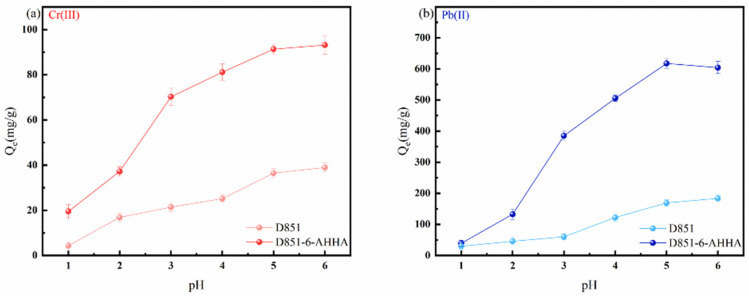
Effect of pH on the adsorption of Cr(III) (**a**) and Pb(II) (**b**) by D851 resin and D851-6-AHHA resin.

It has been demonstrated that the pH value of the solution is a crucial factor influencing the adsorption process of the adsorbent, thus affecting the ionization degree and adsorption sites of heavy metal ions in the solution [35]. To explore the impact of the pH value on adsorption, batch adsorption experiments of Cr(III) and Pb(II) were conducted in the pH range of 1.0 to 6.0. The results are shown in Figure 3a,b, from which the adsorption capacity of the modified D851-6-AHHA resin for Cr(III) and Pb(II) in solution significantly surpasses that of the original resin. When the pH value increased from 1.0 to 5.0, the adsorption capacity of the D851-6-AHHA resin for Cr(III) and Pb(II) increased accordingly. Specifically, the adsorption capacity of Cr(III) rose from 36.45 mg/g to 91.50 mg/g, and the adsorption capacity of Pb(II) increased from 190.63 mg/g to 611.92 mg/g. Therefore, compared with the D851 resin, the adsorption capacity of the D851-6-AHHA resin for Cr(III) and Pb(II) increased by 2.51 times and 3.21 times, respectively, and the adsorption capacity of the D851-6-AHHA resin for Pb(II) was 6.69 times that of Cr(III), indicating that its affinity for Pb(II) was greater than that of Cr(III). On the contrary, when the pH exceeded 5.0, there was a slight decrease in the adsorption capacity of Pb(II), while the adsorption capacity of Cr(III) remained essentially unchanged. The pH value of the solution influenced the surface charge of the D851-6-AHHA resin [36]. At low pH values, a significant amount of H^+^ ions occupied the binding sites of the D851-6-AHHA resin, resulting in the protonation of its functional groups. This protonation resulted in a positive charge on the resin’s surface, leading to electrostatic repulsion and a reduction in its chelating capacity for Cr(III) and Pb(II) [37]. As the pH of the solution continued to increase, the protonation tendency of the binding sites on the D851-6-AHHA resin decreased, resulting in a slight increase in its adsorption capacity for Cr(III) and Pb(II). Therefore, the optimum pH value for the adsorption process was determined to be 5.0.

#### 3.2.2. Adsorption Kinetics Study 

The effect of adsorption time on the adsorption of heavy metal ions by D851-6-AHHA resin is shown in Figure S1, and to further analyze the adsorption kinetics of Cr(III) and Pb(II) in water by the D851-6-AHHA resin, the quasi-first-order and quasi-second-order dynamic equations were employed to fit the experimental data, as expressed in Equations (3) and (4), respectively [38,39]:

ln(Q_e_ − Q_t_) = lnQ_e_ − k_1_t (3)

(4) $$\frac{t}{Q_{t}}=\frac{t}{Q_{e}}+\frac{1}{K_{2}Q_{e}^{2}}$$

where Q_e_ (mg/g) and Q_t_ (mg/g) represent the adsorption amounts of Cr(III) and Pb(II) by the D851-6-AHHA resin at the adsorption equilibrium and at a specific time (t), respectively, K_1_ (g·mg^−1^·min^−1^) denotes the adsorption rate constant for the quasi-first-order kinetic equation, and K_2_ (g·mg^−1^·min^−1^) represents the adsorption rate constant for the quasi-second-order kinetic equation [40,41,42].

**Figure 4:**
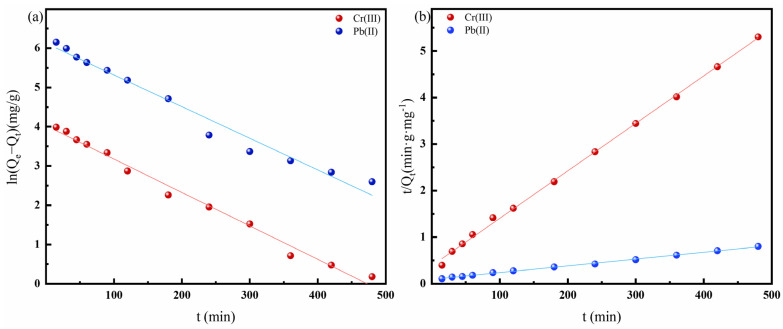
Quasi-first-order fitting (**a**) and quasi-second-order fitting (**b**) of adsorption of Cr(III) and Pb(II) by D851-6-AHHA resin.

The results are depicted in Figure 4a,b and Table 1. The correlation coefficient of the quasi-second-order kinetic model (R^2^ ≥ 0.9980) surpassed that of the quasi-first-order kinetic model (R^2^ ≥ 0.9742). Under the quasi-first-order kinetic model, there was a significant disparity between the experimentally measured adsorption capacity (Q_exp_) and the calculated one (Q_cal_). In contrast, under the quasi-second-order kinetic model, the two values were similar. Therefore, the quasi-second-order kinetic model could better elucidate the adsorption process of the D851-6-AHHA resin, wherein heavy metal ions underwent chemisorption on its surface [43,44].

#### 3.2.3. Isotherm Study

The adsorption isotherm model elucidates the distribution of solute in the liquid phase between the adsorbent and the solvent when the adsorption reaches the equilibrium [45]. In order to gain a deeper understanding of the adsorption mechanism, Langmuir and Freundlich’s adsorption isotherm models were utilized to analyze the experimental data. The corresponding Equations (5) and (6) were as follows [46]:

(5) $$\mathrm{Langmuir}:\;Q_{e}=\frac{K_{L}Q_{m}C_{e}}{1+K_{L}C_{e}}$$

(6) $$\mathrm{Freundlich}:\;Q_{e}=K_{F}C_{e}^{\frac{1}{n}}$$

where Q_e_ (mg/L) is the adsorbed amount at the adsorption equilibrium, Q_m_ (mg/g) is the saturated adsorbed amount in the monolayer, C_e_ (mg/L) is the adsorption equilibrium concentration of heavy metal ions in the solution, K_L_ (L/mg) is the Langmuir equilibrium constant indicating the strength of the adsorption capacity, K_F_ (mg^1−1/n^·L^1/n^·g^−1^) and n are two empirical constants, K_F_ denotes the magnitude of the adsorption capacity of the adsorbent, and 1/n denotes the state of the distribution of the energy as well as the inhomogeneity of the position of the adsorbent [47].

**Figure 5:**
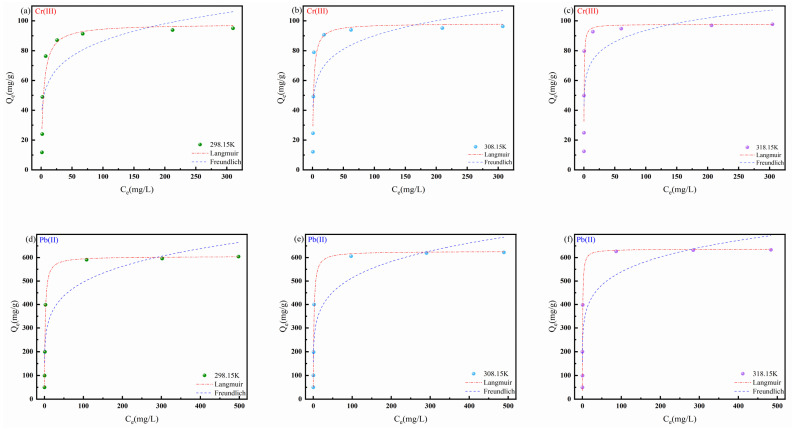
Fitted curves of the quasi-first-order kinetic model (**a**–**c**) and quasi-second-order kinetic model (**d**–**f**) for Cr(III) and Pb(II) adsorption by D851-6-AHHA resin.

The Langmuir and Freundlich adsorption isotherms for the adsorption of heavy metal ions by the D851-6-AHHA resin at various temperatures are depicted in Figure 5a–f, and the original adsorption isotherms are presented in Figure S2. The corresponding equilibrium constants and correlation coefficients (R^2^) are listed in Table 2. It can be seen that the R^2^ value of the Langmuir isotherm surpasses that of the Freundlich isotherm, indicating that it is suitable for describing the adsorption process of Cr(III) and Pb(II) by the D851-6-AHHA resin and the process is monolayer adsorption. Additionally, at a temperature of 298.15 K, the theoretical values of Q_m_ for Cr(III) and Pb(II) of the D851-6-AHHA resin were 97.84 and 615.81 mg/g, respectively. These values exceeded the maximal adsorption capacities of Cr(III) and Pb(II) of the D851 resin, which were 41.08 and 193.83 mg/g, respectively. This discrepancy suggests that the (-C(=O)NHOH) group exhibited a higher affinity for heavy metal ions [48]. Meanwhile, Table S2 summarized the adsorption properties of various adsorbents [49,50,51,52,53,54,55,56]. Compared with these, the D851-6-AHHA resin developed in this study demonstrated better potential for application.

#### 3.2.4. Adsorption Thermodynamics

Parameters such as the enthalpy change (ΔH, kJ/mol), entropy change (ΔS, J/mol/K), and Gibbs free energy change (ΔG, kJ/mol) were estimated using the Van’t Hoff equation. These parameters were employed to determine the spontaneity and type of adsorption, providing insights into the extent of the change on the surface of the adsorbent [57]. The analysis of these parameters allows for a deeper understanding of the driving forces and feasibility of the adsorption process. The Equations (7)–(9) of adsorption thermodynamics were as follows [58]:

(7) $${\mathrm{InK}}_{D}=\frac{\Delta S}{R}\;-\frac{\Delta H}{\mathrm{RT}}$$

(8) $$K_{D}=\;\frac{\mathrm{Qe}/Q^{\theta }}{\mathrm{Qe}/C^{\theta }}$$

ΔG = ΔH − ΔST (9)

where K_D_ is the standard equilibrium constant, R is the universal gas constant (8.314 J/mol/K), T is the temperature in Kelvin (K), Q_e_ is the amount of adsorption at equilibrium for the initial concentration, C_e_ is the initial concentration, C^θ^ is the standard state of the adsorbate in the liquid taken as 1 mol/L, and Q^θ^ is the standard state of the adsorbent on the surface taken as 1 mol/kg [59].

**Figure 6:**
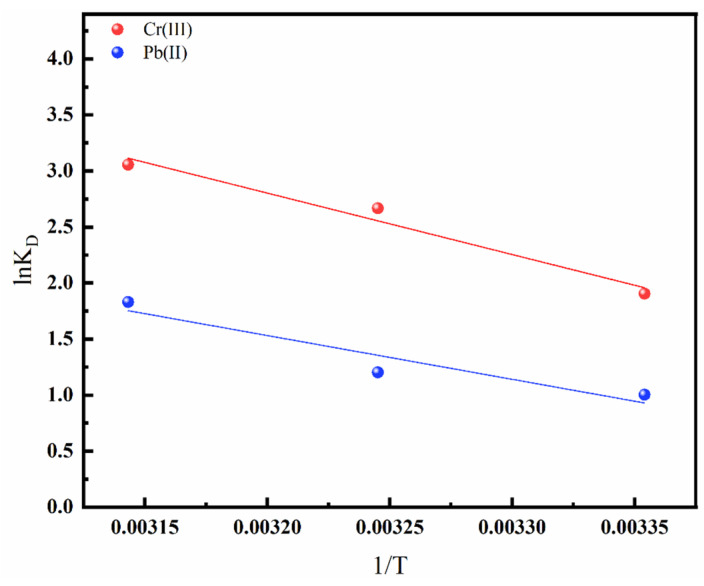
Linear form of adsorption thermodynamic plot.

To understand the impact of the temperature on the adsorption of Cr(III) and Pb(II) by the D851-6-AHHA resin, the data were fitted using the Van’t Hoff equation, and the results are depicted in Figure 6. The relevant parameters including the enthalpy change (ΔH), entropy change (ΔS), and Gibbs free energy change (ΔG) are summarized in Table 3. The enthalpy change (ΔH) values were all positive, indicating that the entire process was an endothermic reaction involving chemical adsorption [60]. The entropy change (ΔS) was also positive, suggesting an increase in the system entropy and an increase in the degrees of freedom [61]. Additionally, the ΔG values were all negative, indicating that the adsorption process was spontaneous. The absolute values of ΔG increased with the increasing temperature, suggesting that higher temperatures favored the adsorption of the resin, which may be related to the diffusion rate of heavy metal ions in the liquid and the solubility of the D851-6-AHHA resin [62].

#### 3.2.5. Reusability of the D851-6-AHHA Resin

**Figure 7:**
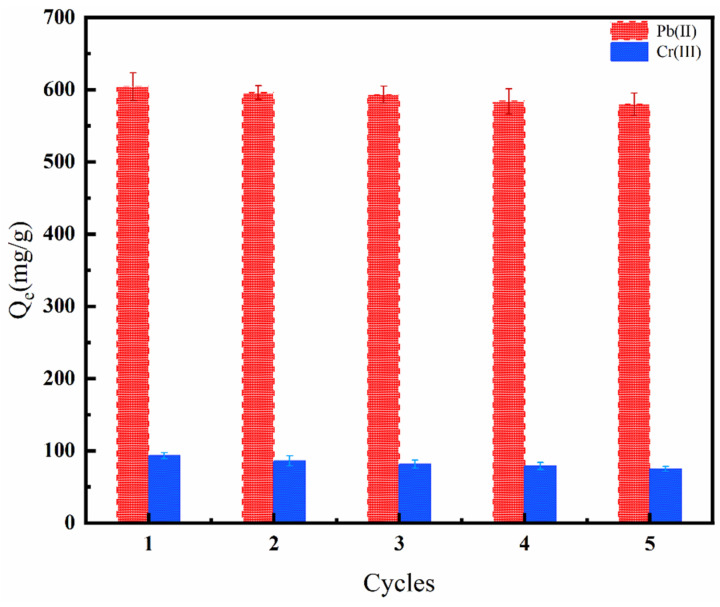
Adsorption and desorption cycles of D851-6-AHHA resin for Cr(III) and Pb(II).

Recycling was employed as an evaluation index for assessing the adsorption performance of the adsorbent. Adsorption–desorption experiments were conducted over five cycles, with desorption carried out using 0.5 mol/L HCl. The results are depicted in Figure 7. Despite a decrease in Cr(III) and Pb(II) adsorption by the D851-6-AHHA resin after five cycles, the adsorption capacities remained at 74.80 mg/g and 583.82 mg/g, respectively. This suggests that HCl effectively desorbed Cr(III) and Pb(II) without compromising the integrity of the (-C(=O)NHOH) group. Meanwhile, the FT-IR spectrum analysis and SEM analysis of D851-6-AHHA resin after five experimental cycles were carried out. As shown in Figures S4 and S5, it can be observed that the peak corresponding to each functional group remains almost unchanged, indicating the high mechanical strength of the resin. The experimental findings highlighted the excellent regeneration performance and stability of the D851-6-AHHA resin, indicating its potential for industrial applications [63].

#### 3.2.6. Performance of D851-6-AHHA Resin in Fixed-Bed Post Systems

The removal ability of the D851-6-AHHA resin for heavy metals in water was investigated by fixed-bed adsorption experiments. The penetration curve was constructed by plotting the ratio of the concentration of heavy metal ions in the effluent of the column (C_t_) to the initial concentration of the influent solution (C_0_) (C_t_/C_0_) against time. The equilibrium point of the column was determined as the point where C_t_/C_0_ reached 95% [64]. According to the “Electroplating Pollutant Emission Standards”, the emission limits for Cr(III) and Pb(II) were set at 1.5 and 1.0 mg/L, respectively, which served as the penetration points for this experiment. As shown in Figure S6a,b, the equilibrium point for the dynamic adsorption of Cr(III) and Pb(II) with the D851-6-AHHA resin was reached at 648 and 666 min, respectively. Meanwhile, the dynamic adsorption of Cr(III) and Pb(II) by the D851-6-AHHA resin reached their respective penetration points at 54 and 72 min. Therefore, 135 mL and 180 mL of Cr(III) and Pb(II) wastewater of comparable concentrations could be treated with 0.60 g of the D851-6-AHHA resin and meet the discharge standard, confirming the significant adsorption performance of the D851-6-AHHA resin on heavy metal ions.

### 3.3. Adsorption Mechanism

**Figure 8:**
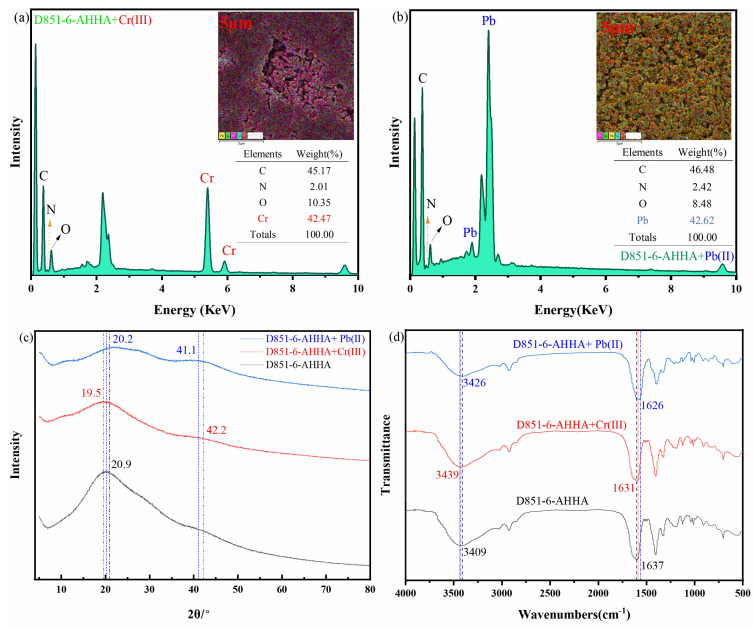
SEM-EDS images of Cr(III) (**a**) and Pb(II) (**b**) adsorbed on D851-6-AHHA resin, XRD patterns (**c**) and FT-IR patterns (**d**) before and after adsorption of Cr(III) and Pb(II) on D851-6-AHHA resin.

SEM-EDS, XRD, FT-IR, and XPS analyses were employed to elucidate the chelating mechanism of the D851-6-AHHA resin for Cr(III) and Pb(II). The findings of the relevant analyses are outlined below. SEM-EDS images of Cr(III) and Pb(II) adsorbed on the D851-6-AHHA resin are presented in Figure 8a,b, revealing mass fractions of 42.47% and 42.62% for Cr(III) and Pb(II), respectively. These results confirm the effective adsorption of these metal ions onto the D851-6-AHHA resin. Figure 8c illustrates the XRD results of the D851-6-AHHA resin before and after the adsorption of Cr(III) and Pb(II) to identify the amorphous and crystalline structure of the resin. Following the adsorption, a broad peak emerged in the D851-6-AHHA resin, shifting from 2θ = 20.9° to lower angles of 2θ = 19.5° and 20.2°. This shift indicates the successful adsorption of the metal ions onto the resin [65]. Moreover, distinct peaks corresponding to the amorphous structure appeared around 2θ = 42.2° and 41.1°, suggesting a significant influence of these heavy metal ions on the reduction of the resin’s crystalline structure [66]. FT-IR spectral analysis was conducted to explore the interaction between the metal ions and the (-C(=O)NHOH) group, as depicted in Figure 8d. For the D851-6-AHHA resin, the peaks of the C=O band in the (-C(=O)NHOH) group shifted to lower wavelengths (from 1637 to 1631 and 1626 cm^−1^) following the adsorption of Cr(III) and Pb(II), respectively [67]. Additionally, the characteristic peaks of the O-H band shifted to higher wavelengths (from 3409 to 3439 and 3426 cm^−1^) after the adsorption of Cr(III) and Pb(II), respectively. These results indicate the participation of the C=O and O-H bands of the (-C(=O)NHOH) group in the coordination of Cr(III) and Pb(II) [68].

**Figure 9:**
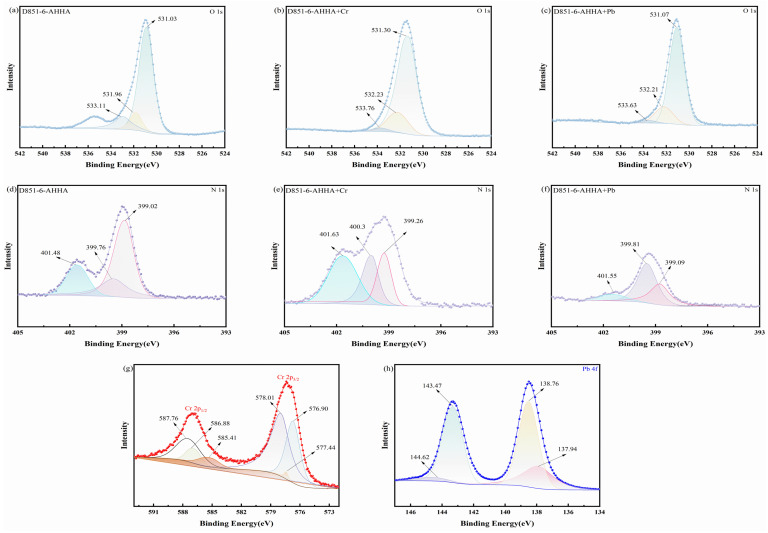
XPS spectra of high-resolution O 1s (**a**–**c**), N 1s (**d**–**f**), Cr 2p (**g**), and Pb 4f (**h**) of D851-6-AHHA resin before and after adsorption of Cr(III) and Pb(II).

XPS analysis plays a crucial role in analyzing the chemical state of elements and, therefore, can be used to investigate changes before and after the adsorption of heavy metal ions [69]. As indicated in Figure S7 and Table S3, both Cr(III) and Pb(II) are effectively adsorbed onto the D851-6-AHHA resin [70]. In Figure 9a, the O 1s spectrum of the D851-6-AHHA resin exhibits three overlapping peaks at binding energies of 531.03 eV, 531.96 eV, and 533.11 eV, corresponding to C=O, C-OH, and N-O bonds, respectively. Upon the adsorption of Cr(III) and Pb(II), the binding energies of both the C-OH and C=O peaks were increased. This phenomenon was attributed to the chelating effect of the (-C(=O)NHOH) group, which donates electrons to the oxygen atom (C=O peak: from 531.03 eV to 531.30 eV and 531.07 eV; C-OH peak: from 531.96 eV to 532.23 eV and 532.21 eV) [71]. In Figure 9d, the N 1s spectra of the D851-6-AHHA resin before and after the adsorption of Cr(III) and Pb(II) exhibit three overlapping peaks at binding energies of 399.02 eV, 399.76 eV, and 401.48 eV, corresponding to the C=N peak, the O=C-N peak, and the N-H peak, respectively. Upon the adsorption of Cr(III) and Pb(II), the binding energy of the C=N bond shifted from 399.02 eV to 399.26 eV and 399.09 eV, while the N-H bond shifted from 401.48 eV to 401.63 eV and 401.55 eV, respectively [72]. The increase in the binding energy indicates that the (-C(=O)NHOH) group in the D851-6-AHHA resin plays an important role in the adsorption of Cr(III) and Pb(II), reflecting the strong chelating power of this group for heavy metal ions [20]. XPS analysis was conducted to investigate the adsorption of Cr(III) and Pb(II) by the D851-6-AHHA resin, as illustrated in Figure 9g,h. Upon the adsorption of Cr(III) and Pb(II), the binding energies at the Pb 4f_7/2_ and Pb 4f_5/2_ spectral bands were measured to be 138.76 eV and 143.47 eV, respectively. These peaks correspond to the structure (-C(=O)NHO-Pb) [73]. Additionally, the binding energy at the Pb 4f_7/2_ spectral band at 137.94 eV was attributed to (-CH_2_N-(CH_2_COO-Pb)_2_), indicating that certain carboxyl groups on the surface of the D851-6-AHHA resin remained unaltered and were not involved in the graft modification process with 6-AHHA. The Cr 2p spectrum comprises complex multiple peaks, with the Cr 2p_3/2_ spectrum primarily composed of three peaks located at 576.90 eV, 577.44 eV, and 578.01 eV, while the Cr 2p_1/2_ spectrum corresponds to three peaks located at 585.41 eV, 586.88 eV, and 587.76 eV, respectively. Specifically, the peak at 578.01 eV in the Cr 2p_3/2_ spectrum corresponds to (-C(=O)NHO-Cr), while the peak at 576.90 eV is attributed to (-CH_2_N-(CH_2_COO-Cr)_2_) [74]. Therefore, the change in the atomic binding energy indicates that the D851-6-AHHA resin exhibits an excellent bonding ability with heavy metal ions. The above analyses show that the -OH portion of the (-C(=O)NHOH) moiety produces stretching bands in the adsorption of Cr(III) and Pb(II), and the binding energy of the C-OH bond will increase [75]. Meanwhile, the N atom in the (-C(=O)NHOH) group is a key site that affects the adsorption of heavy metal ions, the N-H group located at 401.48 eV shows an especially obvious binding power for Cr(III) and Pb(II), and the N-H band is shifted after the adsorption process is completed [76]. In conclusion, the D851-6-AHHA resin contains the (-C(=O)NHOH) group, (-C(=O)NH-) group, and (-CH_2_N-(CH_2_COOH)_2_) group. This composition allows the resin to establish a multielement ring structure with heavy metal ions in the water body, showcasing a strong adsorption performance.

**Scheme 2:**
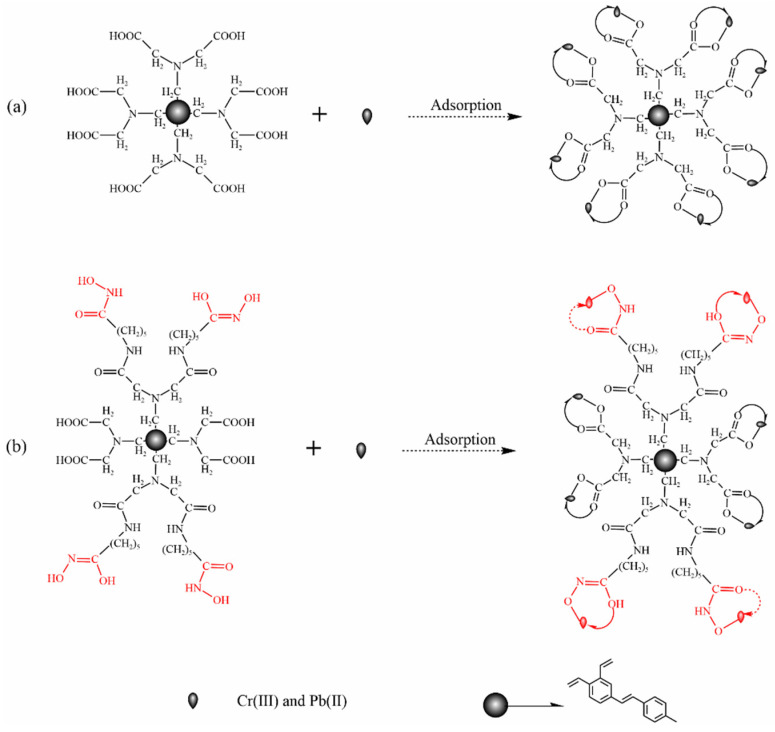
Possible coordination structures of (**a**) D851 and (**b**) D851-6-AHHA resin with Cr(III) and Pb(II), respectively.

Based on the characterization analysis of the D851-6-AHHA resin before and after adsorption of Cr(III) and Pb(II), it was evident that the chelating (-C(=O)NHOH) group of the D851-6-AHHA resin adsorbed heavy metal ions, as depicted in Scheme 2. Additionally, the unreacted (-CH_2_N-(CH_2_COOH)_2_) group on the D851-6-AHHA resin was also capable of forming complexes with (-C(=O)NHOH) in the wastewater. When the isohydroxamic acid group adsorbed Cr(III) and Pb(II), it typically exhibited a distinct chelating morphology, often forming a stable five-membered ring structure with Cr(III) and Pb(II) through a bidentate ligand of the O,O-coordination system [77]. The stability of this five-membered ring structure was approximately seven times greater than that of the carboxylate tetradentate ring structure. Additionally, according to the theory of hard and soft acids and bases (HSAB), it was understood that Cr(III) and Pb(II) belonged to hard and intermediate acids, respectively [78]. Therefore, they readily formed coordination bonds with hard or intermediate bases containing O, N, and S. The D851-6-AHHA chelating resin, which contained the isohydroxamic acid group, featured double ligands of hydroxyl and oxime groups. The presence of these ligands enabled the isohydroxamic acid group to chelate with various metal ions and form stable five-membered ring complexes, thereby exhibiting an excellent adsorption performance.

## 4. Conclusions

In this experiment, a novel chelating resin, D851-6-AHHA, was successfully synthesized by grafting 6-amino-N-hydroxyhexanamide (6-AHHA) onto the (-CH_2_N-(CH_2_COOH)_2_) group of the D851 resin, in order to remove Cr(III) and Pb(II) from wastewater. The influences of the pH, temperature, initial concentration of metal ions, and reaction time on the adsorption of Cr(III) and Pb(II) from water were investigated through batch adsorption experiments utilizing the D851-6-AHHA resin. The findings revealed that the adsorption kinetics of the D851-6-AHHA resin were in accordance with the quasi-second-order kinetic model, while the adsorption isotherms followed the Langmuir model. These outcomes suggest that the resin’s adsorption mechanism predominantly involves chemisorption and monolayer adsorption. Moreover, the overall adsorption process was spontaneous and endothermic. Compared to the D851 resin, the adsorption capacity of the D851-6-AHHA resin for Cr(III) and Pb(II) increased by 2.51 and 3.21 times, respectively. Furthermore, the dynamic adsorption process of Cr(III) and Pb(II) by the D851-6-AHHA resin was investigated via fixed-bed adsorption experiments, showing that 0.60 g of the D851-6-AHHA resin could treat 135 mL and 180 mL of Cr(III) and Pb(II) wastewaters with comparable concentrations. The competitive adsorption of Cr(Ⅲ) and Pb (Ⅱ) by D851-6-AHHA resin was studied by competitive adsorption experiment. As shown in Figure S3, the priority adsorption order of heavy metal ions by D851-6-AHHA resin was Pb(II) > Cr(III). The five-cycle test showed that the D851-6-AHHA resin has an excellent performance. The adsorption mechanism of D851-6-AHHA was explored through SEM-EDS, XRD, FT-IR, and XPS analyses. These investigations revealed the presence of diverse active functional groups in the D851-6-AHHA resin, including (-C(=O)NHOH) and (-COOH) groups, which exhibit effective metal ion adsorption capabilities. In particular, the -CONHOH group forms polycyclic ring structures with Cr(III) and Pb(II). In summary, this experiment successfully synthesized a novel resin with a notable adsorption capacity, demonstrating its promising potential for the remediation of heavy metal ions in water.

## Figures and Tables

**Table 1:** Kinetic parameters of adsorption of Cr(III) and Pb(II) by D851-6-AHHA resin.

Adsorbent	Adsorbates	Q_exp_ (mg/g)	Pseudo-First-Order			Pseudo-Second-Order		
			Q_cal_ (mg/g)	K_1_ (min^−1^)	R^2^	Q_cal_ (mg/g)	K_2_ (g∙mg^−1^∙min^−1^)	R^2^
D851-6-AHHA	Cr(III)	91.50	56.15	0.0085	0.9884	98.04	0.0003	0.9986
	Pb(II)	611.92	456.88	0.9742	0.9742	689.66	0.00002	0.9980

**Table 2:** Adsorption isotherm parameters.

Adsorbent	Adsorbates	T (K)	Langmuir Constants			Freundlich Constants		
			Q_m_	K_L_	R^2^	1/n	K_F_	R^2^
D851-6-AHHA	Cr(III)	298.15	97.84	0.2733	0.9579	0.1805	37.6713	0.6539
		308.15	98.23	0.6037	0.9916	0.1525	44.5854	0.6622
		318.15	101.67	0.7263	0.9402	0.1228	53.1289	0.6899
	Pb(II)	298.15	615.81	0.5754	0.9947	0.1823	214.1348	0.8342
		308.15	642.32	0.6218	0.9876	0.1827	221.5111	0.8306
		318.15	636.07	1.4201	0.9935	0.1596	258.6712	0.7916

**Table 3:** Thermodynamic parameters for the adsorption of Cr(III) and Pb(II) by D851-6-AHHA resin.

Adsorbent	Adsorbates	ΔS (J/mol/K)	ΔH (KJ/mol)	R^2^		ΔG (KJ/mol)	
					298.15 K	308.15 K	318.15 K
D851-6-AHHA	Cr(III)	169.14	45.58	0.9727	−4.85	−6.54	−8.23
	Pb(II)	116.67	32.48	0.9073	−2.30	−3.47	−4.64

## Data Availability

Data are contained within the article and the Supplementary Materials.

## Supplementary Materials

The following supporting information can be downloaded at: https://www.mdpi.com/article/10.3390/polym16141966/s1, Table S1. The surface properties of D851 and D851-6-AHHA resin; Figure S1. Effect of adsorption time on adsorption of heavy metal ions by D851-6-AHHA resin; Figure S2. The adsorption isotherms of D851-6-AHHA resin towards Cr(III) and Pb(II) with different temperatures; Table S2. The comparison of the adsorption capacity of the different adsorbents; Figure S3. Study on Adsorption of D851-6-AHHA Resin in Cr(III) and Pb(II) Systems; Figure S4. FT-IR spectra of D851-6-AHHA resin before and after five cycles; Figure S5. SEM of D851-6-AHHA resin and regenerated resin after desorption of Cr(III) and Pb(II). (a) D851-6-AHHA resin, (b) Resin after desorption of Cr(III), (c) Resin after desorption of Pb(II); Figure S6. Dynamic adsorption curves (a,b) of Cr(III) and Pb(II) by D851-6-AHHA resin; Figure S7. The XPS spectra of the D851-6-AHHA resin before and after the adsorption of Cr(III) and Pb(II); Table S3. The atomic concentration (%) data of XPS spectra for the resin with heavy metal ions.
