# Supplementary material for: Preparation of 6-Amino-N-hydroxyhexanamide-Modified Porous Chelating Resin for Adsorption of Heavy Metal Ions
Source: Polymers (Basel). 2024 Jul 9;16(14):1966. doi: 10.3390/polym16141966 (PMC11281118; doi:10.3390/polym16141966)
Supplement: Supplementary file 1 [file polymers-16-01966-s001.zip › polymers-3032064-supplementary.pdf]

polymers

MDPI

Supplementary Material

Preparation of 6-Amino-N-Hydroxyhexanamide-Modified Porous Chelating Resin for Adsorption of Heavy Metal Ions

Shaomin Liu [1,2,*], Zihan Wang [1], Mingyi He [1] and Jinglin Zhu [1]

[1] School of Earth and Environment, Anhui University of Science and Technology, Huainan 232001, China; zihanwang1999@163.com (Z.W.); 18116779416@163.com (M.H.); jlzhu@aust.edu.cn (J.Z.)
[2] State Key Laboratory of Mining Response and Disaster Prevention and Control in Deep Coal Mines (Anhui University of Science and Technology), Huainan 232001, China
* Correspondence: shmliu1@163.com

1. Experiments

1.1. Preparation of 6-amino-N-hydroxyhexanamide (6-AHHA)

The synthesized 6-amino-N-hydroxycaproamide was purified (recrystallized in an ethanol-water system). The standard curve was set by ultraviolet spectrophotometry, and then the content of 6-amino-N-hydroxycaproamide was calculated by the standard curve (Zhang et al., 2010). The yield was about 83.59%.

The molar ratio of carboxyl groups in the iminodiacetic acid moiety of D851 resin to the -NH_2 groups of 6-amino-N-hydroxycaproamide during synthesis was 0.78 : 1.0. This ratio facilitated a more complete amidation reaction between the two species. Based on the adsorption capacity of the cation exchange resin (GB/T 8114-2008), the carboxyl group content in the iminodiacetic acid moiety within 1.0 g of dried D851 resin was determined to be 0.4621 g. Simultaneously, the mass of the pure product in a 50 ml solution of 6-amino-N-hydroxyhexanamide was measured as 12.20 g. The grafting rate, calculated using the following equation, was found to be 63.42%.

$$G = \frac{n_1 - n_2}{n_3}$$

where G is the grafting rate, n_1 (mol) and n_2 (mol) were the contents of 6-amino-N-hydroxycaproamide before and after grafting. Further, the n_3 (mol) value represented the molar mass of carboxyl groups in iminodiacetic acid groups in the dried D851 resin.

1.2. Bath Adsorption Experiments

1.2.1. Effect of pH

0.10 g and 0.05 g of pre-treated D851-6-AHHA resin were measured and placed into separate 250 mL conical flasks. Subsequently, 50 mL of 200 mg/L Cr(III) solution and 100 mL of 400 mg/L Pb(II) solution were added to the respective conical flasks. The pH of each solution was adjusted to the range of 1.0 to 6.0 using dilute hydrochloric acid and dilute sodium hydroxide solutions at room temperature. The flasks were then placed in a thermostatic oscillator set at 25°C and rotated at 120 r/min for 8 hours to investigate the impact of pH on the adsorption efficiency.

1.2.2. Adsorption Kinetics

For adsorption kinetic studies, 0.10 g and 0.05 g of pre-treated D851-6-AHHA resin were weighed separately and placed into 250 mL conical flasks. Subsequently, 50 mL of 200 mg/L Cr(III) solution and 100 mL of 400 mg/L Pb(II) solution were added to their respective conical flasks. The pH of each solution was adjusted to 5.0 at room temperature. These flasks were then placed in a thermostatic oscillator set at 25°C. When specific time intervals (15, 30, 45, 60, 90, 120, 180, 240, 300, 360, 420, 480 minutes) were reached, 5.0 mL of solution sample was collected. These samples were diluted as necessary and analyzed

for heavy metal concentration using ICP-AES to investigate the effect of time on the adsorption efficiency.

1.2.3. Adsorption Isotherms

For adsorption isotherm studies, 0.10 g and 0.05 g of pre-treated D851-6-AHHA resin were weighed separately and placed into 250 mL conical flasks. A series of Cr(III) solution concentrations (25, 50, 100, 160, 200, 250, 400, 500 mg/L) and Pb(II) solution concentrations (25, 50, 100, 160, 200, 400, 600, 800 mg/L) were prepared by adding 50 mL of Cr(III) solution and 100 mL of Pb(II) solution, respectively, to each conical flask. The initial pH of each solution was adjusted to 5.0 using dilute hydrochloric acid and dilute sodium hydroxide. The conical flasks were then placed in a thermostatic water bath shaker set to 298.15 K and shaken at 120 r/min until equilibrium was reached. After reaching equilibrium, the samples were removed from the solution, diluted appropriately, and the adsorption capacity was determined using ICP-AES. These isothermal adsorption experiments were repeated at temperatures of 298.15 K, 308.15 K, and 318.15 K to investigate the temperature effect on adsorption capacity.

1.2.4. Competitive Adsorption

The competitive adsorption of D851-6-AHHA resin in Cr(III) and Pb(II) systems was investigated. 0.075 g of D851-6-AHHA resin was weighed and transferred into a 250 mL conical flask. 300 mg/L stock solutions of Cr(III) and Pb(II) were prepared, and 75 mL of each solution was added to the conical flask containing the resin. The pH of the mixed solution was adjusted to 5.0. The flask was placed on a shaking table, and the competitive adsorption of D851-6-AHHA resin on the metal ions was analyzed after equilibration.

2. Results and Discussion

2.1. Characterization

Table S1. The surface properties of D851 and D851-6-AHHA resin.

Adsorbebts	BET Surface Area ($m^2 \cdot g^{-1}$)	Pore Volume ($cm^3 \cdot g^{-1}$)	Average Pore Size (nm)
D851	9.204	0.068	35.169
D851-6-AHHA	14.057	0.086	30.533

2.2. Bath Adsorption Experiments

2.2.1. Adsorption Kinetics Study

The presented figure depicted the effect of time on the adsorption process of D851-6-AHHA resin. It was evident from the figure that the adsorption capacity of Cr(III) and Pb(II) by D851-6-AHHA resin exhibited a rapid increase from 0 to 60 minutes. This observed phenomenon could be attributed to the formation of a significant concentration difference between the heavy metal ions in the solution and the surface of D851-6-AHHA resin at the onset of the adsorption reaction. Meanwhile, the substantial number of adsorption sites on the resin's surface facilitated the rapid binding of heavy metal ions to it, resulting in a significant increase in the adsorption capacity. Approximately 300 minutes later, adsorption equilibrium was attained, leading to a plateau in the adsorption curve. Notably, the adsorption capacity of D851-6-AHHA resin for Pb(II) surpassed that for Cr(III), potentially attributed to the greater affinity of the (-C(=O)NHOH) group for Pb(II).

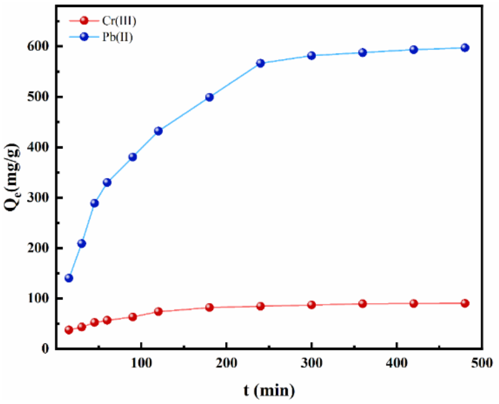

Figure S1. Effect of adsorption time on adsorption of heavy metal ions by D851-6-AHHA resin.

2.2.2. Isotherm Study

In this experiment, the adsorption effect of D851-6-AHHA resin on Cr(III) and Pb(II) was investigated at different temperatures and different initial concentrations (25-800 mg/L). The results depicted in Fig. S2(a-b) indicated that at low initial concentrations of heavy metal ions, nearly complete adsorption onto the D851-6-AHHA resin was achieved. During this phase, Cr(III) and Pb(II) extensively interacted with the adsorption sites on the resin's surface, leading to a rapid increase in adsorption capacity. As the concentration gradually increased, the rate of adsorption growth slowed until reaching an equilibrium state. Meanwhile, it was observed from the figure that the adsorption amount of D851-6-AHHA resin increased with the gradual increase in temperature, indicating that the increase in temperature was favorable to the adsorption of the resin.

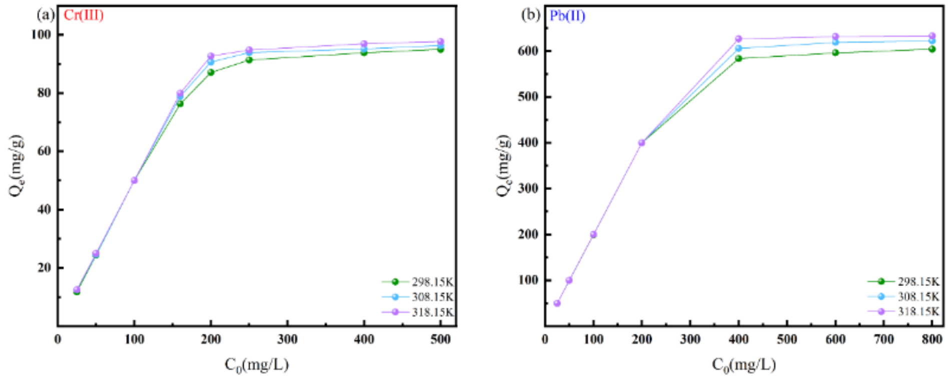

Figure S2. The adsorption isotherms of D851-6-AHHA resin towards Cr(III) and Pb(II) with different temperatures.

Table S2. The comparison of the adsorption capacity of the different adsorbents.

Adsorbent	pH	Temperature	Adsorption capacity ($mg \cdot g^{-1}$)		Ref.
			Cr(III)	Pb(II)	
Magnetic resin microspheres	5.5	298.15K	-	219.30	(Li et al., 2019)
Activated carbon	6.0	298.15K	-	171.00	(Wang et al., 2018)
Amberlite Sulfonic acid group	5.0	298.15K	88.00	-	(Kocaoba et al., 2005)
Lewatit S: Sulfonic acid group with cross linked polystyrene matrix	3.5	298.15K	77.00	-	(Gode et al., 2006)
Thiacalixarene-loaded resin	6.0	293.15 K	-	74.90	(Hu et al., 2010)
Leucodon sciuroides	6.0	298.15 K	-	207.20	(Ucarli et al., 2020)
Sulfonated styrene/acrylonitrile	6.0	298.15 K	48.00	-	(Kanwal et al., 2005)

D851 resin	5.0	298.15 K	36.45	190.63	This work
D851-6-AHHA	5.0	298.15 K	91.50	611.92	This work

3.2.3. Competitive Adsorption

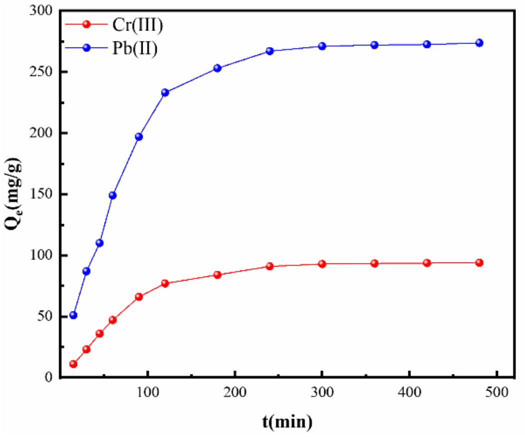

Figure S3. Study on Adsorption of D851-6-AHHA Resin in Cr(III) and Pb(II) Systems.

Fig. S3 demonstrated that D851-6-AHHA resin exhibited high adsorption capacities for both Cr(III) and Pb(II) at pH 5.0, reaching 87.41 mg/g and 271.98 mg/g, respectively. However, the presence of both heavy metal ions concurrently resulted in a lower adsorption capacity for each individual ion compared to their adsorption in isolation. This suggested a competitive adsorption phenomenon between Cr(III) and Pb(II). Notably, the adsorption capacity for Pb(II) remained significantly higher than that of Cr(III) under these conditions. Therefore, the preferential adsorption order of heavy metals by D851-6-AHHA resin could be established as Pb(II) > Cr(III).

3.2.4. Reusability of the D851-6-AHHA Resin

The FT-IR spectra of the D851-6-AHHA resin after five cycles of experiments are depicted in Fig. S4, and it can be observed that the peaks corresponding to each functional group remain almost unchanged. Additionally, the structure of the D851-6-AHHA resin appears to be unaltered, as shown in Fig. S5, indicating the high mechanical strength of the resin.

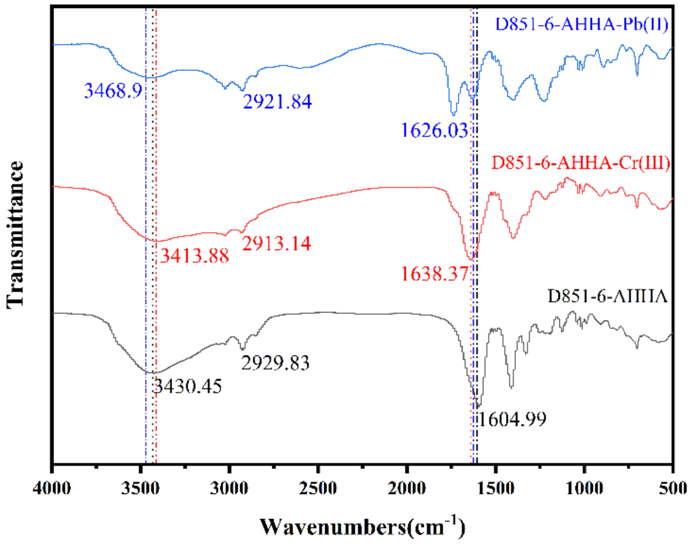

Figure S4. FT-IR spectra of D851-6-AHHA resin before and after five cycles.

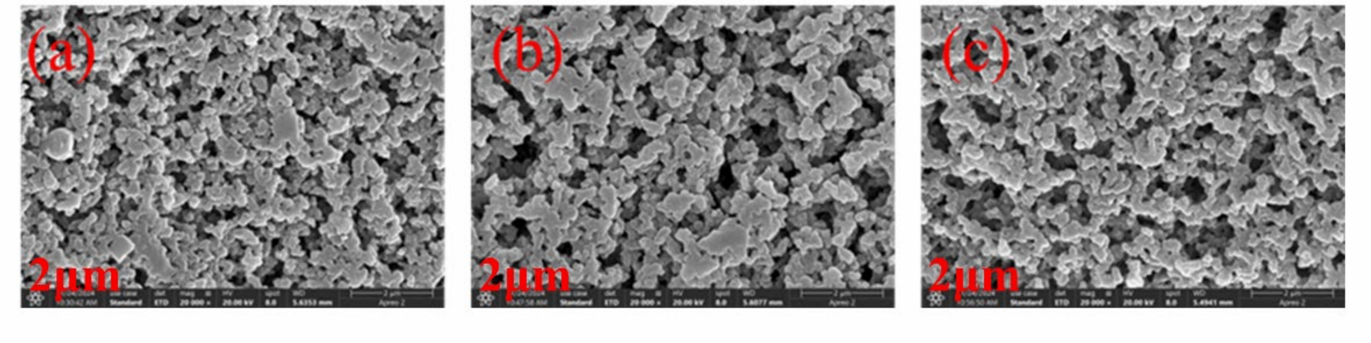

Figure S5. SEM of D851-6-AHHA resin and regenerated resin after desorption of Cr(III) and Pb(II). (a) D851-6-AHHA resin, (b) Resin after desorption of Cr(III), (c) Resin after desorption of Pb(II).

3.2.5. Performance of D851-6-AHHA Resin in Fixed-bed Post Systems

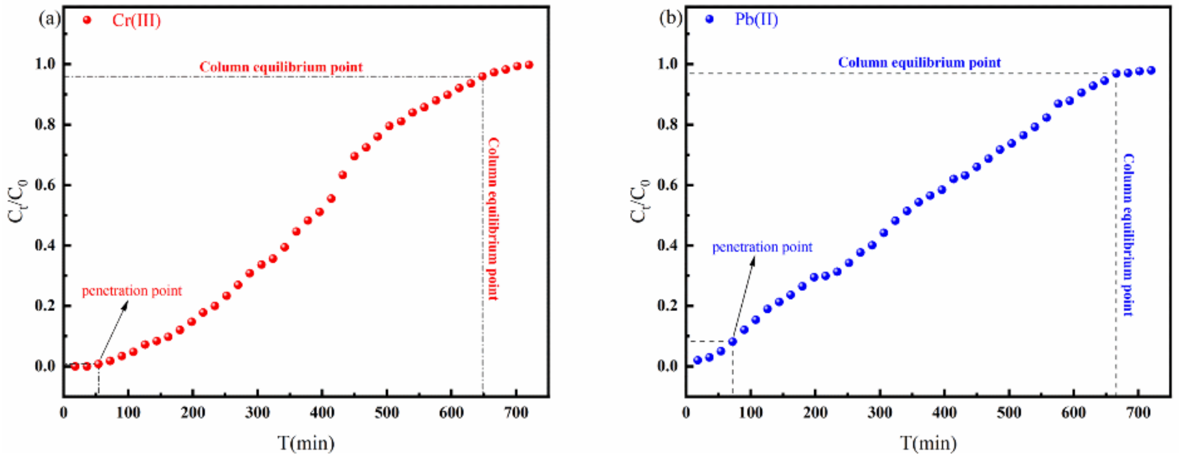

Figure S6. Dynamic adsorption curves (a-b) of Cr(III) and Pb(II) by D851-6-AHHA resin.

2.3. Adsorption Mechanism

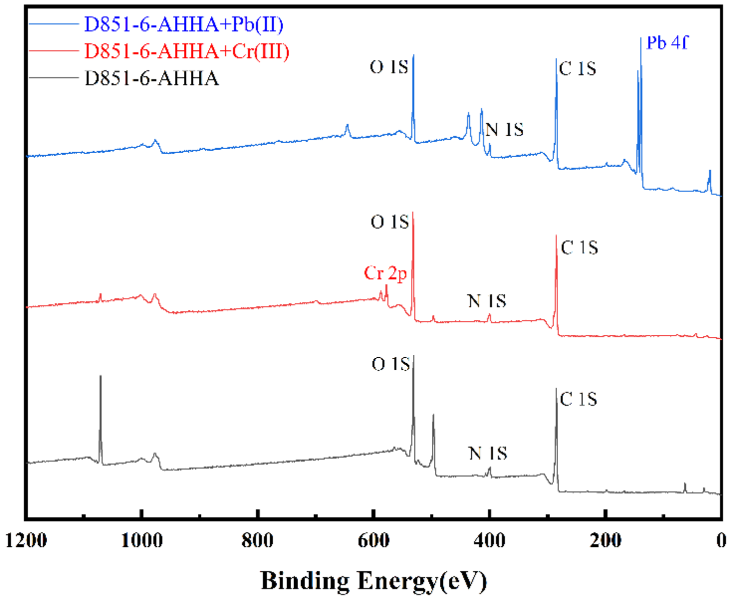

Figure S7. The XPS spectra of the D851-6-AHHA resin before and after the adsorption of Cr(III) and Pb(II).

Table S3. The atomic concentration (%) data of XPS spectra for the resin with heavy metal ions.

Materials	Atomic (%)				
	C 1s	O 1s	N 1s	Cr 2p	Pb 4f
D851-6-AHHA	70.47	24.82	4.71	-	-
D851-6-AHHA+Cr	65.62	26.05	5.72	2.61	-
D851-6-AHHA+Pb	70.11	21.28	5.24	-	3.37

References

1. Gode F, Pehlivan E, 2006. Removal of chromium(III) from aqueous solutions using Lewatit S 100: The effect of pH, time, metal concentration and temperature. Journal of Hazardous Materials, 136(2): 330-337. DOI:10.1016/j.jhazmat.2005.12.021.
2. Hu X, Li Y, Wang Y, et al, 2010. Adsorption kinetics, thermodynamics and isotherm of thiacalix[4]arene-loaded resin to heavy metal ions. Desalination, 259(1): 76-83. DOI:10.1016/j.desal.2010.04.032.
3. Kanwal F, Imran M, Mitu L, et al., NaN/NaN/NaN. Removal of Chromium(III) Using Synthetic Polymers, Copolymers and their Sulfonated Derivatives as Adsorbents. Journal of Chemistry, 9: 621-630. DOI:10.1155/2012/857579.
4. Kocaoba S, Akcin G, 2005. Removal of chromium (III) and cadmium (II) from aqueous solutions. Desalination, 180(1): 151-156. DOI:10.1016/j.desal.2004.12.034.
5. Li Z, Wang Z, Wang C, et al., 2019. Preparation of magnetic resin microspheres M-P(MMA-DVB-GMA) and the adsorption property to heavy metal ions. Applied Surface Science, 496: 143708. DOI:10.1016/j.apsusc.2019.143708.
6. Ucarli O, Yayintas O T, Engin M S, et al., 2020. Investigation of Competitive and Noncompetitive Adsorption of Some Heavy Metals Ions on *Leucodon sciuroides* (Hedw.) Schwägr. Langmuir, 36(28): 8265-8271. DOI:10.1021/acs.langmuir.0c01403.
7. Wang N, Zhai Y, Yang Y, et al, 2018. Electrostatic assembly of superwetting porous nanofibrous membrane toward oil-in-water microemulsion separation. Chemical Engineering Journal, 354: 463-472. DOI:10.1016/j.cej.2018.08.019.
8. Zhang B, Zhong H, 2010. Determination of hydroxamic acids by direct spectrophotometry of colored complex in acidic solution. Research on Chemical Intermediates, 36(5): 495-501. DOI:10.1007/s11164-010-0160-3.
